# Open-Cell Tizr-Based Bulk Metallic Glass Scaffolds with Excellent Biocompatibility and Suitable Mechanical Properties for Biomedical Application

**DOI:** 10.3390/jfb11020028

**Published:** 2020-05-01

**Authors:** Van Tai Nguyen, Xavier Pei-Chun Wong, Sin-Mao Song, Pei-Hua Tsai, Jason Shian-Ching Jang, I-Yu Tsao, Che-Hsin Lin, Van Cuong Nguyen

**Affiliations:** 1 Department of Mechanical Engineering, National Central University, Chung-Li 32001, Taiwan; nvtai87@gmail.com; 2 Department of Mechanical Engineering, Can Tho University, 3/2, Can Tho 900000, Viet Nam; nvcuong@ctu.edu.vn; 3 School of Biomedical Engineering, Taipei Medical University, Taipei 110, Taiwan; s0925135546@yahoo.com.tw; 4 Institute of Materials Science and Engineering, National Central University, Chung-Li 32001, Taiwan; bear82112760103@gmail.com (S.-M.S.); peggyphtsai@gmail.com (P.-H.T.); evauseonly@yahoo.com.tw (I.-Y.T.); 5 Department of Mechanical and Electro-Mechanical Engineering, National Sun Yat-Sen University, Kaohsiung 80424, Taiwan; chehsin@mail.nsysu.edu.tw

**Keywords:** bulk metallic glass, scaffold, biomaterials, cell viability, calcium deposition, porosity, mechanical property

## Abstract

A series of biocompatible high-porosity (up to 72.4%) TiZr-based porous bulk metallic glass (BMG) scaffolds were successfully fabricated by hot pressing a mixture of toxic element-free TiZr-based BMG powder and an Al particle space holder. The morphology of the fabricated scaffolds was similar to that of human bones, with pore sizes ranging from 75 to 250 μm. X-ray diffraction patterns and transmission electron microscopy images indicated that the amorphous structure of the TiZr-based BMG scaffolds remained in the amorphous state after hot pressing. Noncytotoxicity and extracellular calcium deposition of the TiZr-based BMG scaffolds at porosities of 32.8%, 48.8%, and 64.0% were examined by using the direct contact method. The results showed that the BMG scaffolds possess high cell viability and extracellular calcium deposition with average cell survival and deposition rates of approximately 170.1% and 130.9%, respectively. In addition, the resulting TiZr-based BMG scaffolds exhibited a considerable reduction in Young’s moduli from 56.4 to 2.3 GPa, compressive strength from 979 to 19 MPa, and bending strength from 157 MPa to 49 MPa when the porosity was gradually increased from 2.0% to 72.4%. Based on the aforementioned specific characteristics, TiZr-based BMG scaffolds can be considered as potential candidates for biomedical applications in the human body.

## 1. Introduction

TiZr bulk metallic glasses (BMGs) have been fabricated to replace metallic materials in bioimplant applications because of the excellent biocompatibility, corrosion behavior, and mechanical properties of BMGs [1,2,3]. Li et al. [3] revealed that a cell survival rate of 99% ± 5% can be achieved for Ti_40_Zr_10_Cu_36_Pd_14_ BMG. Furthermore, a large number of cells were observed on the surface of this BMG in addition to excellent adhesion behavior. Huang et al. [4] demonstrated that TiZr-based amorphous alloys exhibit excellent biocompatibility in male New Zealand white rabbits in vivo. The obtained results indicated excellent osteoinduction. No local inflammation occurred near the amorphous alloy, and the density of the newly generated spongy bone near the amorphous alloy interface was similar to that of the spongy bone near the cortical bone. However, a large mismatch in the mechanical behavior, especially Young’s modulus, between the implant made of the TiZr-based BMG and human bones, causes implant loosening, which results in stress shielding [5,6,7,8]. Moreover, the cytotoxicity of elements, such as Cu, Ni, Al, and Be, used to fabricate the TiZr-based BMGs was considered because of their toxic ion release. Niinomi et al. [9] discovered that Ni causes allergic reactions, especially in the female population, which increases with the increase in the amount of Ni. Huang [10,11] mentioned that large concentrations of Cu ions are harmful to the D1 cell, which reduces biocompatibility and cell viability.

Biocompatible BMGs should be capable of interacting with tissues in the human body without causing harm to the body. Numerous factors should be considered from the perspective of the long-term immersion of biocompatible implants in the human body. These include (1) responses of the human body to the implant or cell-biological activity of the implant, (2) degradation of the BMG under corrosive activity in the body environment, and (3) nonallergic and toxic constituents originating from the metal ions or particles of the implant released into the human body [12]. Therefore, TiZr-based BMGs designed using nontoxic elements such as Nb, Ta, Zr, Si, Sn, Pd, In, Sr, B, Ca, and Mg have been investigated as promising BMGs for implantable medical devices and orthopedic applications.

Porous structural alloys (such as cellular structures) with a structure similar to the structure of the human bone are being developed for use in bioimplant and clinical applications. The interconnected porous structure of the alloys allows new bone tissue cells to infiltrate into the implant and form excellent logical fixation with the surrounding tissue [13]. Thus, increasing the porosity for the bioimplant is necessary for regenerating bones and avoiding stress shielding in the human body. Based on the density of the foam, the mechanical properties of a porous implant can be controlled by adjusting its relative porosity. In our previous studies [14,15,16], we have successfully estimated the Young’s modulus and compressive strength of the Zr-based and TiZr-based open-cell BMG scaffolds by controlling the relative density using the Gibson and Ashby model. 

The TiZr-based BMG with the nominal composition of Ti_42_Zr_35_Si_5_Ta_3_Co_12.5_Sn_2.5_ (in atomic percentage) was designed in our previous studies as the pre-alloy ingots [16,17]. The TiZr-based BMG showed a high glass-forming ability (GFA) with glass transition (T_g_) and crystallization (T_x_) temperatures are approximately 758 and 853 K, respectively, together with a large supercooled liquid region $\left(SLR,\;\Delta T=T_{x}-T_{g}\approx 95\;K\right)$ and a considerably low liquidus temperature of 1153 K (or 880 °C). This proved that the TiZr-based metallic glass possesses the excellent thermal properties required to form BMGFs. Although the chemical composition contains of Co element are considered as a toxic element, the Co alloys have been developed for biomedical applications [18,19,20]. Catelas et al. [18] and Pérez-Maceda et al. [20] mentioned that CoCr wear particles obtained from a metal–metal hip simulator did not contain Co, and the chromium oxide particles were mostly generated by wear of the passivation layer covering the implant surface in the wear of metal–metal hip implants. Thus, the Co element has been used as a friendly element for long-term immersion in the human body to design the biomedical device. In parallel, the addition of Co element into the TiZr-based BMG can significantly improve the GFA due to its low melting point, as summarized in Table 1.

Biocompatible BMG scaffolds with high porosity, fabricated by using nontoxic elements and a suitable space holder, have played important roles for biomedical applications in the human body. Bonding-force interface between amorphous alloy particles in the porous sample can be attributed to involving to the added spacer into the matrix powder. Thermal energy can rapidly homogeneously transfer to whole amorphous alloy particles during hot pressing. In this study, Al particles with a high thermal conductivity of approximately 240 (W/mK) at room temperature are considered as spacer particles to improve the bonding-force interface between TiZr-based amorphous alloy particles, resulting in enhancing the porosity of the porous sample. We used Al particles with sizes in the range 75–250 μm, and mixed them with the toxic-element-free Ti_42_Zr_35_Ta_3_Si_5_Co_12.5_Sn_2.5_ amorphous alloy powder. Then, we combined the TiZr-based powder and the Al space holder by controlling the volume fraction of Al particles to fabricate the TiZr-based BMG scaffolds with porosities ranging from 2.0% to 72.4%. The characteristics of the prepared TiZr-based BMG scaffolds, including morphology, crystallinity, biocompatibility, and mechanical properties, were characterized and discussed.

## 2. Materials and Methods 

### 2.1. Sample Fabrication

TiZr-based powders with a chemical composition of Ti_42_Zr_35_Si_5_Ta_3_Co_12.5_Sn_2.5_ (in atomic percentage) were prepared using the atomization process under argon atmosphere. The atomized alloy powders with particle sizes less than 25 μm were assumed as completly amorphous and used as the matrix powder for preparing BMG scaffolds. Bulk scaffolds with various porosities ranging from 2.0% to 72.4% were designed by mixing the Al spacer particles and the TiZr-based matrix powder with different volume fractions. Based on the results obtained using hot pressing in our previous studies [14,15,16], the stress, temperature, and holding time were set to 300 MPa, 520 °C (temperature within the supercooled liquid region), and 300 s, respectively. Then, the Al spacer particles were removed from the prepared scaffolds in a 2 M NaOH warm solution (approximately 70 °C). Finally, the porous samples were cut into some small parts to assess the existence of the Al spacer particles inside the TiZr-based BMG scaffolds through X-ray diffraction and scanning electron microscopy for cross-sectional surface at different positions of the porous samples.

### 2.2. Real Porosity 

The real porosity of the TiZr-based BMG scaffolds was obtained by using the Archimedes method and verified using statistical averaging calculation over six measurements. Before the samples were immersed into 2 M NaOH warm solution, the original volume of the samples including the volume of BMG powder, Al spacer particles and voids was firstly calculated by mass deviation of the sample in air and in water (as Equation (1)). Then, the porous samples (after removing Al spacer particles) were immersed into water to determine the volume of pores and voids through amount of penetrating water into the porous samples (as Equation (2)). The real porosity was calculated as the ratio between original volume and the volume of pores and voids, as in Equation (3)

(1) $$V_{1}=\frac{m_{1}-m_{2}}{D}$$

(2) $$V_{2}=\frac{m_{4}-m_{3}}{D}$$

(3) $$Real\;porosity\;\left(\%\right)=\frac{V_{2}}{V_{1}}\times 100$$

where *V*_1_ and *V*_2_ are original volume of the sample, and volume of pores and voids, respectively; *m*_1_ and *m*_2_ are the weight of the sample in air and water before removing Al spacer particles, respectively; *m*_3_ and *m*_4_ are the weight of the porous sample in air and the weight of the porous sample containing of penetrating water, respectively; *D* is density of water.

### 2.3. Morphology

The morphology and the pore size of the hot-pressed scaffolds were observed through scanning electron microscopy (SEM, Field-emission Scanning Electron Microscopy Inspect F50, Thermo Fisher Scientific Inc, Bartlesville, OK, USA, operated at 20 keV). From the SEM images, the pore sizes were estimated considering the diameter of the connecting cavity because the cells were not spherical close cells. The average pore size of the TiZr-based BMG scaffolds was calculated by conducting 100 measurements of cell images from cross-sectional SEM micrographs of each type of the TiZr-based BMG scaffolds. 

### 2.4. Structure Analysis

The amorphous structure of the TiZr-based BMG scaffolds was characterized through X-ray diffraction (XRD, D8 advance X-ray diffraction, Bruker Corporation, Bremen, Germany, operated at 40 kV) and transmission electron microscopy (TEM, JEOL-JEM2100 High Resolution STEM, Japan Electron Optics Laboratory Co., Ltd., Tokyo, Japan, operated at 200 keV). The foil specimens used in the TEM examination were sliced from the hot-pressed foam by using a dual beam-focused ion beam system (FIB, FEI Versa 3D High-Resolution Dual-Beam Focus-Ion-Beam System, Thermo Fisher Scientific Inc, operated at 30 kV). 

### 2.5. Biocompatibility Tests

To measure the cell viability (3-(4,5-dimethylthiazol-2-yl)-2,5-diphenyltetrazolium bromide (MTT) assay) and extracellular matrix calcium deposition (ARS) of MG63 cells, the direct contact method was used. For the cell viability test, TiZr-based BMG scaffolds were first placed into a 24-well plate; then, 5000 cells/well of the MG63 cells were seeded into the well for incubation. After a 3-d incubation, the samples were transferred to a new 24-well plate to detect cell viability, which was defined by the number of cells that adhered on the surface of samples. A 10-μL MTT reagent (Invitrogen, Carlsbad, CA, USA) was then added into each well carefully followed by incubation for 3 h. Then, the samples were placed in a new 24-well plate and 100 μL of dimethylsulfoxide was added into each well. Optical density was measured using an enzyme-linked immune-sorbent assay reader at a wavelength of 560 nm (Multiskan FC; Thermo, Waltham, MA, USA). 

Simultaneously, the MG63 cells were selected to perform extracellular matrix calcium deposition for various porosities of the TiZr-based BMG scaffolds. The specimens were placed into a 24-well plate, and the cell culture process was similar to the cell viability test. After 7 days of incubation, the specimens were placed in a new 48-well plate and 4% paraformaldehyde was added. Alizarin red S (ARS) staining dye was used to stain the calcium deposit generated by the MG63 cells. After 40 min, the ARS dye was removed and solubilized with 100 μL of dimethyl sulfoxide for 30 min and then the enzyme-linked immune-sorbent assay reader (SPECTRA MAX 190, Molecular Device, San Jose, CA, USA) with 590 nm wavelength was used to measure the optical density.

Three different measurements were separately conducted and calculated average values for cell viability and extracellular calcium deposition. The results of cell viability and extracellular calcium deposition are presented as mean ± standard deviation. The analysis was performed using SPSS 20. One-way ANOVA analyses of variance followed by post hoc Tukey tests were performed in this study.

### 2.6. Mechanical Properties

The mechanical properties of the TiZr-based BMG scaffolds, including its compressive strength, Young’s modulus, and bending strength were characterized using an MTS 810 universal testing machine at an initial strain rate of 1 × 10^−4^ s^−1^ at room temperature. To prepare specimens, a low-speed diamond saw machine was used to cut the TiZr-based BMG scaffolds in the rectangular shape with dimensions of 2.5 T × 2.5 W × 5 L (±0.1 mm) mm^3^ and 1.5 T × 2 W × 25 L (±0.05 mm) mm^3^ for the compression and the bending tests, respectively. Then, the rectangular specimens were polished carefully with sandpaper grades (#1000, #2000, #4000) to ensure their ends were parallel. The bending strength of the specimens was calculated using the ASTM C1161-13 standard formula for three-point flexure $S=\frac{3PL}{2bd^{2}}$, where *S*, *P*, *L*, *b*, and *d* denote the bending strength (MPa), break force (N), outer (support) span (mm), specimen width (mm), and specimen thickness (mm), respectively [21]. 

## 3. Results and Discussion

### 3.1. Removing Al Spacer Particles

**Figure 1:**
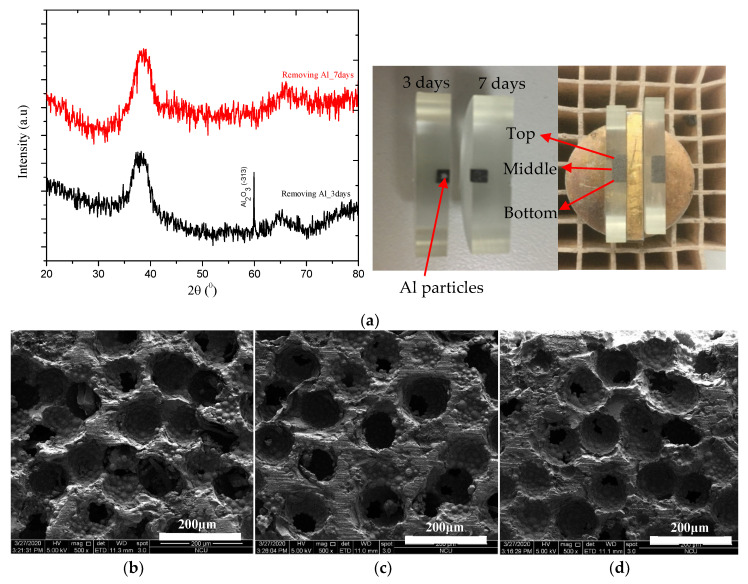
(**a**) XRD patterns of TiZr-based BMG scaffolds with porosity of 72.4% immersed in 2 M NaOH warm solutions for 3 and 7 days. (**b**–**d**) cross-sectional SEM images at different positions (b-top; c-middle; d-bottom) for the scaffold with porosity of 72.4% immersed in 2 M NaOH warm solutions in 14 days.

The scaffolds with porosity of 72.4% were firstly selected to examine the removing Al particle ability by using the 2 M NaOH warm solution $\left(2\mathrm{Al}+2H_{2}O+2\mathrm{NaOH}\to 2{\mathrm{NaAlO}}_{2}+3H_{2}\right)$. The scaffolds were separately immersed into two different beakers of the 2 M NaOH warm solution for 3 days and 7 days, respectively. Then, the scaffolds were rinsed using DI water and dried at room temperature. The results displayed in Figure 1a show that the scaffold immersed into the 2 M NaOH warm solution for 3 days stilled retain some the Al spacer particles at center of the sample with a peak in Al_2_O_3_ located at 2θ of approximately 60° in XRD patterns. In parallel, the XRD pattern and image for the scaffold immersed into the 2 M NaOH warm solution for 7 days indicated that no Al spacer particles can be observed at center of the sample. 

To make sure that the Al spacer particles can be fully removed out, the fabricated scaffolds were immersed in the 2 M NaOH warm solution for 14 days before conducting further analysis. Figure 1b–d presented cross-sectional SEM images for different positions (top–middle–bottom) of the scaffold that the Al spacer particles do not exist inside the scaffold. This proved that the Al spacer particles from the hot-pressed scaffolds can be removed by immersing the scaffold into the 2 M NaOH warm solution for 14 days.

### 3.2. Real Porosity and Morphology of TiZr-Based BMG Scaffolds

The real porosity (vol. %) of the TiZr-based BMG scaffolds was determined as the ratio between original volume of the sample, and volume of pores and voids. The results are summarized in Table 2. BMG scaffolds with porosities of 12.4%, 16.7%, 24.6%, 32.8%, 48.8%, 64.0%, and 72.4% were obtained by hot pressing the mixture of Al spacer particles (size ranging 75–250 μm) and TiZr-based matrix powder with various volume fractions of the spacer (10%, 15%, 20%, 25%, 35%, 45% and 50%). In our previous study [16], the scaffolds fabricated by using NaCl spacer particles just gained at the porosity of 34.8% with suitable mechanical properties in comparison to the mechanical properties of the human bones. It can be assumed that a low thermal conductivity of the NaCl spacer particles, which is approximately 10 (W/mK) at room temperature, negatively affects the interfacial bonding between amorphous alloy particles during hot pressing. The porosity of the biomaterials plays a vital role in bone integration. A high porosity can provide a large interface area between the implant and living tissue, resulting in strong interlocking of surrounding bone tissue with the implant [6]. In addition, the highly porous implant encourages proliferation of the osteoblast-like cells and specific alkaline phosphate activities [22]. The extract contact method was used in our previous study to access the cell viability of MG63 osteoblast-like cells cultured in various precipitate media of TiZr-based BMG scaffolds. The results showed that the cell viability (in %) increased with the increase in the porosity of the TiZr-based BMG scaffold. The TiZr-based BMG scaffold with a porosity of 34.8% provided an average cell viability of 98%. Thus, increasing the porosity of bioimplants is necessary for biocompatible materials, and the Al particle is a promising spacer that can be added into the TiZr-based BMG scaffold to improve the porosity of the implant. 

**Figure 2:**
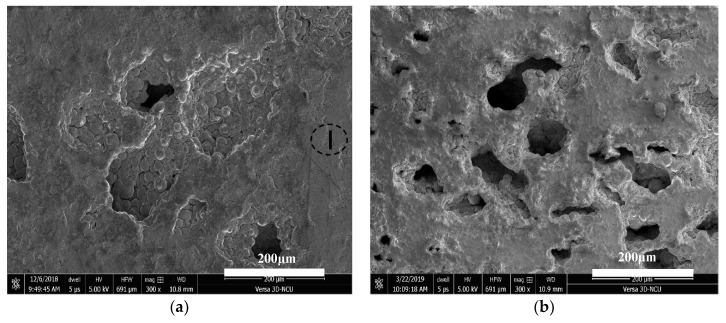

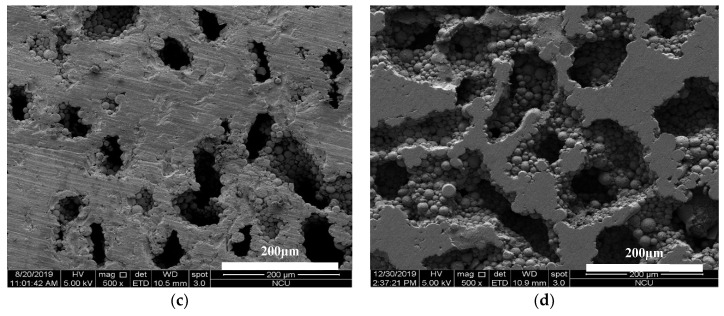
(**a**–**d**) Representative cross-sectional morphology of TiZr-based BMG scaffolds with real porosities of (**a**) 16.7%, (**b**) 32.8%, (**c**) 48.8%, and (**d**) 64.0%.

Human bones can be divided into three major structural groups (based on their pore sizes), namely Haversian/Volkmann’s canals, osteocytic lacunae, and canaliculi. The Haversian canals, with nominal pore sizes ranging from 25 to 335 μm, comprise the main structural group in human bones [23]. Kasten et al. [22] revealed that the minimum required pore size is 50–100 μm for general bone regeneration. Based on the alkaline phosphatase activity, Tsuruga et al. [24] revealed that pore sizes of 212–400 μm result in a high osteoinductive ability and compared the osteocalcium content associated with other pore sizes. Thus, Al spacer particles with sizes in the range 75–250 μm were selected to fabricate the TiZr-based BMG scaffolds. Figure 2a–d presents cross-sectional SEM micrographs of the TiZr-based BMG scaffolds with porosities of 16.7%, 32.8%, 48.8%, and 64.0%, respectively. The micrographs indicated that a relatively homogeneous pore size distribution and pore dimensions approximately the size of the Al spacer particles were obtained. The obtained results proved that the Al spacer particles were suitable for fabricating compact scaffolds with appropriate pore sizes. 

### 3.3. Structure Characterization of TiZr-Based BMG Scaffolds

**Figure 3:**
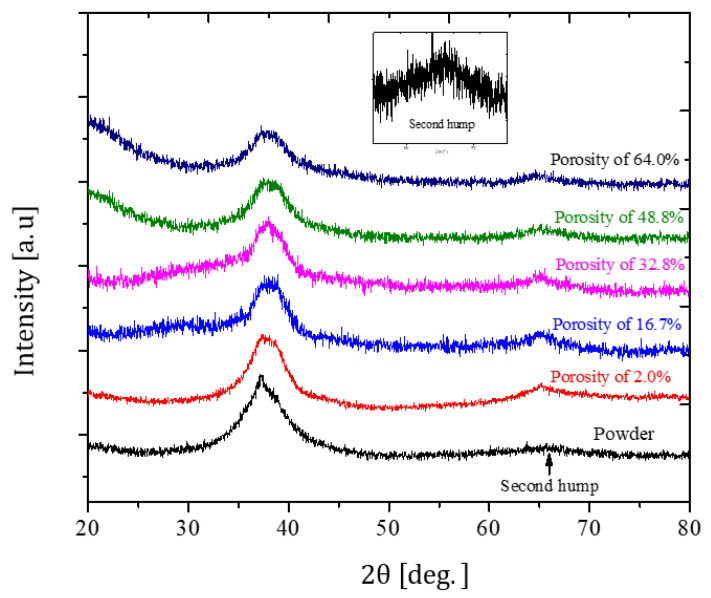
XRD patterns of TiZr-based amorphous powder and BMG scaffolds.

**Figure 4:**
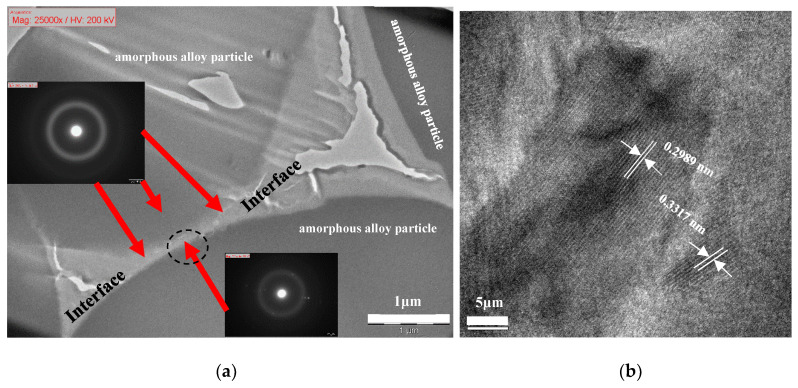
(**a**) Bright-field TEM image of bonding interface indicated by a black line in Figure 2a; (**b**) high resolution TEM image, which was enlarged from the area indicated by the dashed-black oval in Figure 4a.

XRD patterns in Figure 3 indicate that the prepared TiZr-based BMG scaffolds with porosities ranging from 2% to 64% retain their amorphous characteristic after hot pressing, with two distinct normal broad humps within the 2θ ranges of 30°–50° and 60°–70°. This proves that the selected parameter combination of stress (300 MPa), temperature (520 °C), and holding time (5 min) is effective for avoiding the amorphous to crystalline transformation during hot pressing. Moreover, Figure 4a depicts the bright-field TEM image at bonding interfaces of the TiZr-based BMG scaffold with a porosity of 16.7%. The major area of interface between two amorphous alloy particles remained in the amorphous state and exhibited a typical hollow ring of selected area diffraction pattern, as depicted in the inset of Figure 4a, indicating that the BMG scaffold can mostly remain in its amorphous state after hot pressing. Furthermore, a strong bonding–force interface between the amorphous alloy particles, as depicted in Figure 4a, in the TiZr-based BMG scaffolds was obtained after hot pressing. However, a few nano-crystalline phases approximately 5–25 nm in size were embedded in the amorphous interface area of the hot-pressed BMG scaffold (marked by a dashed-black oval in Figure 4a). These nanocrystalline phases contained the main normal alpha-Ti phase with a lattice constant of 0.2989 nm and the minor normal beta-Ti phase with a lattice constant of 0.3317 nm. 

### 3.4. Biocompatibility by Using the Direct Contact Method

#### 3.4.1. Cell Viability

**Figure 5:**
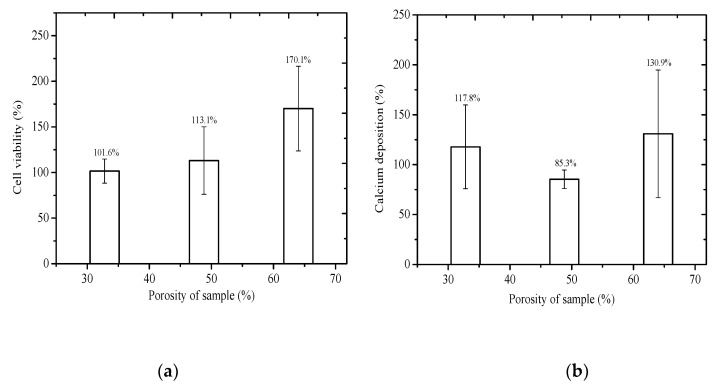
(**a**) Cell viability of MG63 cells cultured on TiZr-based BMG scaffolds with porosities of 32.8%, 48.8%, and 64.0% for 3 days. (**b**) Extracellular matrix calcium deposition of MG63 cells cultured on TiZr-based BMG scaffolds with the three porosities.

Cell viability was determined by the number of healthy cells on the surface of the sample based on total MG63 cells in comparison with the control group. To compare the responses of the control group, the MG63 cells were only exposed to the culture medium and the test samples; the viability of the cell culture in the unexposed group was set at 100%. Figure 5a illustrates the cell viability of the MG63 cells (normalized against the control group) cultured in TiZr-based BMG scaffolds with porosities of 32.8%, 48.8%, and 64.0%. The obtained results indicated that the average values of the cell viability for these prepared samples was higher than 100% in terms of the survival rate of the MG63 cells for all culture mediums. Figure 5a displays that, from the perspective of cell viability, the highest porosity of the foam in this study (64.0%) resulted in a considerably higher cell viability of approximately 170%. Moreover, according to ISO 10993-5 [25], all groups of cell viability can be classified as nontoxic, and cells proliferated.

#### 3.4.2. Extracellular-Matrix Calcium Deposition

The calcium deposit rate of the MG63 cells was evaluated using alizarin red S staining dye and the accumulation of calcium deposit generated by the MG63 cells was compared to the control group. The dark red area, indicating calcification deposition of the MG63 cells, was visualized and captured using an optical microscope (all results are expressed in percentage). Figure 5b displays the average calcium deposition rate of the MG63 cells cultured in various culture media of the TiZr-based BMG scaffolds with porosities of 32.8%, 48.8%, and 64.0%. The calcium deposition rate of the MG63 cells obtained from the culture medium of the sample with a porosity of 64.0% exhibited considerably higher calcification deposits than those cultured in various media of the TiZr-based BMG scaffold with porosities of 32.8% and 48.8%. This implies that a bioimplant with a higher porosity can increase the relative in-growth rate of the human bone. The sample with a porosity of 48.8% showed the lowest calcium deposition rate; however, the calcium deposition rate remained higher than 85%.

### 3.5. Mechanical Properties

#### 3.5.1. Young’s Modulus, Compressive Strength and Bending Strength of Tizr-Based Bmg Scaffolds

**Figure 6:**
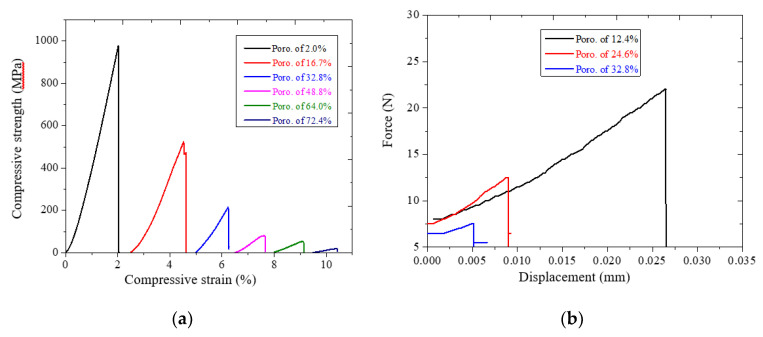
Mechanical properties of TiZr-based BMG scaffolds: (**a**) Stress–strain curves for compression test, (**b**) Force-displacement curves for the three-point bending test.

Figure 6a illustrates the typical compression stress–strain curves of the TiZr-based BMG scaffolds with real porosities ranging from 2.0% to 72.4%. Young’s modulus of the scaffolds was determined by linear fitting of the stress–strain curve [26]. Figure 6 showed that both the Young’s modulus and the compressive strength decreased with the increase in the porosity from 2.0% to 72.4%. The mechanical properties of the TiZr-based BMG scaffolds are summarized in Table 2. The Young’s modulus decreased from 56.4 to 2.3 GPa and the compressive strength from 979 to 19 MPa. The results indicated a porous structural material as opposed to the dense material, which has a representative Young’s modulus of 80–100 GPa for this TiZr-based alloy [12]. Furthermore, Figure 6 b illustrates the relationship between the bending strength and the apparent porosity for the TiZr-based BMG scaffolds with dimensions of 1.5 × 2 × 26 (±0.05, mm) mm^3^; the bending strength decreased from 157 to 49 MPa as the relative porosity increased from 12.4% to 32.8%. Keller et al. reported that in bending tests, adult human compact bone specimens fracture under stresses of 17.8–315 MPa at various positions, such as femoral cortex, tibia, and humerus [27]. The difference in the bending strength values can probably be ascribed to differences in bone density, porosity, mineralization, and microstructure. In comparison to characteristics of the human bones, including Young modulus from 1–35 GPa, compressive strength from 50–238 MPa, and bending strength from 17.8–315 MPa, for the different positions in the outer harder region or the inner softer region [27,28,29], the mechanical properties of the present hot-pressed BMG scaffolds with porosities ranging from 32.8% to 64.0% were similar to the mechanical properties of the human bones. This finding proved that the Al particle used is a suitable spacer for fabricating high-porosity TiZr-based BMG scaffolds in which the high-porosity samples pose a low risk of stress-shielding effect.

#### 3.5.2. Predicting Young’s Modulus and Compressive Strength

The desired Young’s modulus and compressive strength of the porous materials have been approximately predicted using the Gibson and Ashby model [14,15,30], in which the mechanical properties of the porous sample are related to its real porosity (or relative density). The relationships between Young’s modulus and relative density, and between compressive strength and relative density are presented by Equations (4) and (5), respectively,

(4) $$\frac{E}{E_{s}}=C1{\left(\frac{\rho }{\rho_{s}}\right)}^{n1}$$

(5) $$\frac{\sigma_{pl}}{\sigma_{s}}C2{\left(\frac{\rho }{\rho_{s}}\right)}^{n2}$$

where *E*, *σ*_pl_, and *ρ* are the elastic modulus, plateau stress, and density of the porous material, respectively; *E*_s_, *σ*_s_, and *ρ*_s_ are the elastic modulus, compressive strength, and density of the open-cell wall material (i.e., Ti_42_Zr_35_Si_5_Ta_3_Co_12.5_Sn_2.5_); and *C*1, *C*2, *n*1, and *n*2 are constants. According to our previous studies, for the current TiZr-based BMG scaffolds, E_s_ and σ_s_, determined using a nanoindenter, are approximately 112 and 1.34 GPa, respectively; and *ρ*_s_ is approximately 6.08 g/cm^3^.

**Figure 7:**
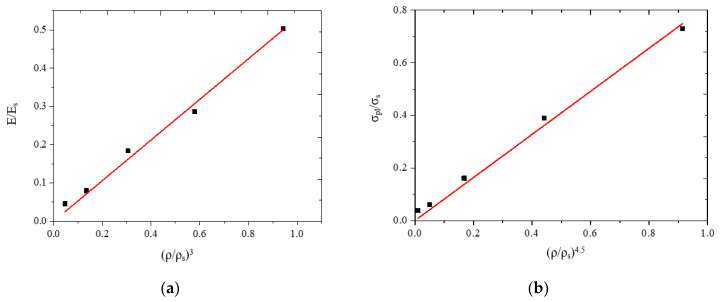
The Gibson and Ashby model was used to assess the relationships between relative BMG scaffold densities and (**a**) Young’s modulus and (**b**) compressive strength.

The values of *C*1, *C*2, *n*1, and *n*2 depended on the structure of the porous material and were related to the bonding strength of the cell walls. The linear fitting lines of the Gibson and Ashby model for Equations (4) and (5) are depicted in Figure 7a–b and are summarized in Table 2. High values of *C*1 (0.53) and *C*2 (0.82) indicated that a strong bonding strength between the amorphous alloy particles can be obtained after hot pressing. The relationship between the relative density and Young’s modulus, E/E_s_ ~0.53 (*ρ*/*ρ*_s_)^3^, and that between relative density and compressive strength, σ_pl_/σ_s_ ~0.82 (*ρ*/*ρ*_s_)^4.5^, have high determination coefficients (R^2^) of 0.995 for both fitting lines. Thus, the dependency of the elastic modulus and the compressive strength of TiZr-based BMG scaffold on the relative density can be accurately predicted using this model.

## 4. Conclusions

A highly porous (up to 64.0%) TiZr-based BMG scaffold was successfully fabricated using Al spacer particles as a suitable substitute for NaCl spacer. In comparison to the scaffold fabricated by using NaCl particles, the porosity of the scaffold produced in here was significantly enhanced from 34.8% to 64.0%. The TiZr-based BMG scaffolds with porosities ranging from 16.7% to 64.0% had suitable pore sizes of 75–250 µm, which are consistent with the pore sizes in human bones. The amorphous structure of the TiZr-based BMG scaffolds was retained after hot pressing. The results of TEM examination presented a strongly bonded interface between the amorphous alloy particles. A bioimplant with a porosity of 64.0% can provide a high cell viability of 170.1% and calcification deposition rate of 130.9%. In addition, the TiZr-based BMG scaffolds with porosity in the range of 32.8% to 64.0% exhibited a Young’s modulus from 20.6 to 5.1 GPa, compressive strength from 216 to 52 MPa, and bending strength of 49 MPa at a porosity of 32.8%, which are comparable with the characteristics of human bones. An excellent combination of high porosity, biocompatibility, and proper mechanical properties renders the high-porosity-TiZr-based BMG scaffolds as a promising structural material for application in biomedical implants. 

## Figures and Tables

**Table 1:** Thermal properties of the TiZr-based bulk metallic glasses (BMGs) with different chemical compositions.

Alloys	$T_{g}\;(K)$	$T_{x}\;(K)$	ΔT	$T_{l}\;(K)$	γ	$\gamma_{m}$	Note
Ti_42_Zr_40_Ta_3_Si_15_	799	898	99	1728	0.355	0.577	base component
Ti_42_Zr_40_Ta_3_Si_7.5_Sn_7.5_	776	904	128	1738	0.360	0.594	+Sn
Ti_42_Zr_42_Ta_3_Si_7.5_Sn_5.5_	763	900	137	1709	0.364	0.607	+Sn
Ti_42_Zr_40_Ta_3_Si_10_Sn_5_	773	910	137	1728	0.364	0.606	+Sn
Ti_42_Zr_42_Ta_3_Si_10_Sn_3_	751	900	149	1703	0.367	0.616	+Sn
Ti_42_Zr_32.5_Ta_3_Si_7.5_Co_15_	777	834	57	1220	0.418	0.730	+Co
Ti_42_Zr_35_Ta_3_Si_10_Co_10_	798	844	46	1191	0.424	0.747	+Co
Ti_42_Zr_35_Ta_3_Si_7.5_Co_12.5_	758	826	68	1199	0.422	0.746	+Co
Ti_42_Zr_35_Ta_3_Si_5_Co_15_	745	817	72	1201	0.420	0.740	+Co
Ti_42_Zr_35_Ta_3_Si_5_Sn_7.5_Co_7.5_	803	874	71	1198	0.427	0.763	+SnCo
Ti_42_Zr_35_Ta_3_Si_5_Sn_5_Co_10_	809	873	64	1212	0.432	0.773	+SnCo
Ti_42_Zr_35_Ta_3_Si_5_Sn_2.5_Co_12.5_	761	842	81	1210	0.437	0.789	+SnCo

**Table 2:** Summary of real porosities and mechanical properties of TiZr-based BMG scaffolds.

Al (vol. %)	Real Porosity (vol. %)	Young’s Modulus (E, GPa)	Compressive Strength (σ, MPa)	Bending Strength (S, MPa)	E/E_s_	σ_pl_/σ_s_	*ρ/ρ_s_*	(*ρ/ρ_s_*)^3^	(*ρ/ρ_s_*)^4.5^
-	0.0	112	1342	-	1	1	1	1	1
Free	2.0	56.4	979	-	0.504	0.730	0.980	0.942	0.914
10	12.4	-	-	157	-	-	-	-	-
15	16.7	32.1	524	-	0.287	0.390	0.834	0.580	0.442
20	24.6	-	-	78	-	-	-	-	-
25	32.8	20.6	216	49	0.184	0.161	0.673	0.304	0.168
35	38.8	8.9	81	-	0.079	0.060	0.513	0.135	0.050
45	64.0	5.1	52	-	0.046	0.039	0.360	0.047	0.010
50	72.4	2.3	19	-	-	-	-	-	-
